# Reading the mind in cartoon eyes: Comparing human versus cartoon emotion recognition in those with high and low levels of autistic traits

**DOI:** 10.1177/0033294120988135

**Published:** 2021-03-09

**Authors:** Gray Atherton, Liam Cross

**Affiliations:** Department of Psychology, 6249 Edge Hill University, Ormskirk, UK

**Keywords:** autism, theory of mind, anthropomorphism, perspective taking, emotion recognition, reading the mind in the eyes, cartoon, facial emotion recognition

## Abstract

People who have a high degree of autistic traits often underperform on theory of mind tasks such as perspective-taking or facial emotion recognition compared to those with lower levels of autistic traits. However, some research suggests that this may not be the case if the agent they are evaluating is anthropomorphic (i.e. animal or cartoon) rather than typically human. The present studies examined the relation between facial emotion recognition and autistic trait profiles in over 750 adults using either a standard or cartoon version of the Reading the Mind in the Eyes (RME) test. Results showed that those scoring above the clinical cut off for autistic traits on the Autism Quotient performed significantly worse than those with the lowest levels of autistic traits on the standard RME, while scores across these groups did not differ substantially on the cartoon version of the task. These findings add further evidence that theory of mind ability such as facial emotion recognition is not at a global deficit in those with a high degree of autistic traits. Instead, differences in this ability may be specific to evaluating human agents.

## Introduction

Autism spectrum condition (ASC) is a neurological condition affecting approximately 2.5% of the population (Kogan et al., 2018). People with ASC show differences in social and communicative processing, and exhibit restricted interests and repetitive behaviors (American Psychiatric Association, 2013). While clinical cases of autism have been documented since the early 1940s (Kanner, 1943), more recently the scientific community has begun to recognize the prevalence of autistic traits throughout the general population (Bolton et al., 1994; Ruzich et al., 2015).

It has been hypothesized that a defining facet of the autism phenotype is a social processing deficit, encompassing both lower-level processing weaknesses relating to facial and emotional recognition, and higher-order processing weaknesses such as difficulties inferring other perspectives and understanding constructs like irony or pretence (Charman et al., 2011). Evidence for the persistence of autistic traits impacting social performance across the spectrum includes studies comparing non-clinical samples with high levels of autistic traits (Piven et al., 1997) to those with lower levels of autistic traits. Research shows that people with high levels of autistic traits show social processing differences in line with clinical samples (Ingersoll, 2010).

One area of continued interest in autism research is understanding differences in face processing, noticeable from infancy and persistent throughout adulthood (Dawson et al., 2005). Both people with a clinical diagnosis of autism and those with high levels of autistic traits have been shown to process faces differently than those with lower levels of autistic traits, including reduced facial recognition, reduced holistic face processing and reduced attention to eyes (Poljac et al., 2013; Tang et al., 2015).

Some research suggests that what underlies this difference may be how rewarding social stimuli is based on whether it is human or non-human (Chevallier et al., 2012). For instance, several studies revealed that those with higher levels of autistic traits possessed an increased preference for and interest in non-human social agents (animals, robots or cartoons) compared to typically human agents (for a review see Atherton & Cross, 2018). Anecdotal evidence for this preference can be found in autobiographic works by the well-known autistic advocates and animal behaviourists Temple Grandin (Grandin & Johnson, 2009) and Dawn Prince-Hughes (Prince-Hughes, 2004). They detail their experiences of social acceptance and discovery after connecting with animals. Similarly, qualitative work has shown autistic people to have a particular affinity for anthropomorphism, particularly in the context of theory of mind (Atherton et al., 2018), and may identify with animals who are similarly viewed as ‘more than human’ (Davidson & Smith, 2009).

Empirical work has also shown that those with higher levels of autistic traits no longer underperformed on perspective-taking tasks when the agents of interest were animals rather than humans (Cross et al., 2019). Cross et al. (2019) showed that young children with a clinical diagnosis of autism and comorbid disorders, such as intellectual disability were better able to correctly recognize emotions from full-face pictures when they were presented in anthropomorphic filters. Silva et al. (2015) found that autistic children were drawn to cartoon faces, while avoidant of human ones, using an approach/avoidance paradigm within an emotion recognition task. Finally, a systematic review by Pennisi et al. (2016) suggested that autistic people were more socially responsive to robots compared to humans, and saw more significant therapeutic gains when interacting with non-human therapeutic partners.

All the work presented so far explored the relationship between Facial Emotion Recognition (FER) and ASC in autistic individuals (the term preferred by members of the autistic community, Kenny et al., 2016). However, as previously discussed, autistic traits are found to be normally distributed across a continuum, with individuals who do not possess a formal diagnosis of autism having similar traits and characteristics. As previously discussed, these individuals show similar difficulties with theory of mind tasks such as perspective-taking. Some work suggests that these differences may also be limited to evaluating human agents. Atherton and Cross (2018) showed that, in line with previous work, those with elevated levels of autistic traits performed more poorly at a non-visual perspective-taking task involving the social faux pas of human agents. These results did not hold when faux pas stories were presented in non-human contexts with animal characters. However, no work has explored whether similar patterns of findings exist for FER. This is a particularly pertinent question due to the neurological and perceptual differences related to ASC and FER abilities.

It is well documented that autistic people show altered processing of faces, such as a hypoactivation of the dedicated neural processing regions the fusiform gyra and fusiform face area (FFA) (Pierce et al., 2001). However, Grelotti et al. (2005) found that an autistic child had typical activation in the fusiform face area (FFA) when viewing a cartoon rather than a human, as did Jung et al. (2016) in response to robot faces, and Whyte et al. (2016) in response to animal faces. With regards to eye-processing, two studies found that autistic children explored the eye areas of animal and cartoon faces, rather than human faces, in line with controls (Grandgeorge et al., 2016; Saitovitch et al., 2013). Some researchers have gone a step further and investigated the effects of processing non-human versus human social stimuli on theory of mind performance. Rosset et al. (2008), for instance, found that autistic children used a typical emotion processing strategy when viewing cartoon rather than human faces in line with controls. Brosnan et al. (2015) even found that autistic adolescents significantly out-performed controls when evaluating emotions from cartoon faces, while performing significantly more poorly when evaluating human stimuli.

Several studies have found face processing differences between those with and without ASC when presented with human faces, and a lack of distinction and even a relative advantage in response to non-human faces (see Atherton & Cross, 2018 for a review). For instance, Brosnan et al. (2015) showed that emotion recognition may be intact or even enhanced when autistic adolescents analyzed cartoon facial expressions. However, the Brosnan study employed a limited set of FER stimuli (for example: excited, kind, sad, surprised, happy, proud) with more straightforward emotions suitable for adolescent samples but not adults, who have developed peak FER performance by early adulthood (Amorim et al., 2019). Thus, it remains unclear whether an increased FER ability with non-human stimuli replicates in an adult sample using suitably complex facial stimuli.

Furthermore, Brosnan et al. (2015) used a stimulus set showing the whole-face, which may have allowed autistic participants to exploit atypical processing strategies to gage emotion, such as focusing on the mouth (Neumann et al., 2006). Research suggests that autistic people and those with high traits were most impaired when focusing on eye regions of the face, while controls were most successful in FER when focusing on eye regions (Joseph & Tanaka, 2003). Thus, it was of interest to utilize an emotion recognition assessment that requires participants to focus on the eyes when performing FER.

To test whether adults were more responsive to a non-human version of a task testing eye-specific FER, we modified the reading the mind in the eyes (RME) to contain cartoon versions of the eye stimuli, and compared adults with high and low autistic trait levels on their performance. In study 1, participants with elevated levels of autistic traits) and participants with low levels of autistic traits were compared on the original RME test. It was hypothesized that those with higher levels of autistic traits would perform more poorly on the standard version of the RME than those with lower levels of autistic traits. In study 2, a novel ‘cartoonized' version of the RME referred to as the Cartoon Reading the Mind in the Eyes (CRME) was developed and a new sample scoring within the same cut-off points was tested. It was hypothesized that, unlike study 1, those with higher levels of autistic traits would no longer underperform on the CRME.

## Study 1

### Methods

#### Participants

Students from a university in the Southwest region of the United States participated in this study (n = 388, 307 females, *M*age = 21.62, age range 17-50, 28.5% White, 28.5% Asian, 25.6% Hispanic, 12.4% Black, 4.5% Other, with an average of several years of college completion). Participants were recruited from an online research platform and received course credit for participation as well as the chance to win one of twenty $20 Amazon gift cards. There were no exclusion criteria for this study. The sample size was not pre-specified at the design stage, and a one-month recruitment window was set with the aim to recruit as many people as possible. This study received approval from the university's Institutional Review Board.

#### Design and procedure

This study employed a between-groups design with one subject variable: Autism Spectrum Quotient (AQ) (Baron-Cohen et al., 2001) score. Participants were later divided into one of two groups (High/Low AQ trait) based on their AQ scores, using pre-defined cut-offs derived from the original Baron-Cohen et al. (2001) study. The dependent variable was emotion recognition as measured by the RME.

This study ran online using Qualtrics (Qualtrics, Provo, UT, USA). After providing consent, participants gave their gender, age, and level of educational attainment. Following this, they completed the RME, followed by the AQ. This order of assessment was employed to control for the carry-over of negative self-appraisals in individuals who endorsed a large frequency of items about autistic traits (as is suggested by Skorich et al., 2016; Yang & Baillargeon, 2013). Following the completion of both the RME and the AQ, participants read a debriefing statement.

For the RME individuals were shown 32 pictures of eyes and asked to pick which emotion the eyes portrayed out of four options (one correct while the other three were foils). These were presented one at a time with the order randomized. To avoid issues with verbal comprehension and the RME (Peterson & Miller, 2012), participants were encouraged when they didn't understand a word to hover their cursor over each of the four emotion words. Doing this superimposed the Oxford dictionary definition of that word, along with a sentence using the word, above the answer choice. As the RME has previously been associated with verbal IQ (Peterson & Miller, 2012), it was essential to include a reference guide for participants to minimize the effect of verbal ability on performance.

The AQ is composed of 50 items which measure levels of agreement with statements concerning personal characteristics and behaviors. It consists of five factors – Social Skills, Attention to Detail, Attention Switching, Communication, Imagination (Hoekstra et al., 2011; James et al., 2016; Kloosterman et al., 2011). Participants received the AQ in the original format. In the AQ, they chose the corresponding response (concerning an autistic trait) which best described their level of agreement using a 4-point scale ranging from strongly disagree and strongly agree. Half of the items required reverse scoring. A higher AQ score corresponds to a higher prevalence of autistic trait endorsement.

### Results

Both the AQ (KS(388) = .07, p = < .001) and the RME (KS(388) = .128, p = < .001) data violated the normality assumption. No outliers were excluded, and non-parametric statistics were used. A non-parametric correlation established the expected negative relationship between AQ and RME scores (rs = −.19, n = 388, p < .001) suggesting that lower scores on the AQ indeed correlated with higher scores on the RME.

High and Low AQ groups were then formulated based on previous research (Baron-Cohen et al., 2001) The High AQ group included every participant who scored within the clinical range of 32 and above on the AQ, which included 10 participants (2.57% of the total sample, in line with current prevalence levels; Kogan et al., 2018). As this group consisted of the ten individuals with the highest-scoring AQ, a matched Low AQ group was then formulated comprising of the 10 participants with the lowest AQ scores. The use of a Low AQ control group was developed to compare those with low vs high levels of autistic traits and follows previous research excluding the middle group of AQ scorers (Almeida et al., 2013; Bayliss & Tipper, 2005; Cooper et al., 2013).

An independent-samples Mann Whitney U test was then performed to explore whether there was a significant difference in RME scores between the High and Low AQ groups. Those in the High AQ group scored significantly worse on the RME than the Low AQ group U = 76.5, Z = 2.02, p = .043, r = 0.45. See Table 1 for the descriptive statistics.

**Table 1.** Descriptive statistics for the AQ total and the RME proportion correct for Study 1.

	AQ				RME			
Group type	M	SD	Mdn	Range	M	SD	Mdn	Range
High AQ	34.6	2.76	33.8	32–39	.72	.10	.75	.58–.86
Low AQ	8.4	1.58	8.75	6–10	.80	.07	.80	.69–.89

### Discussion

In line with our hypothesis that those with higher levels of autistic traits would perform more poorly on the RME than those with lower levels of autistic traits, Study 1's results indicated that the High AQ group underperformed on the RME compared to the Low AQ group. This finding is in line with previous studies that found a significant difference between RME performance amongst people with and without ASC (for a review see Peñuelas-Calvo et al., 2018), as well as studies which showed that these effects persisted regardless of gender, as in Baron-Cohen et al. (2015). It is of interest to note that compared to other studies such as Baron-Cohen et al. (2015), effect sizes were smaller in the present study. It may be that by including the definitions of words on the testing page and allowing ease of access to them through hovering the pointer, participants were less dependent on verbal ability which have been shown to hamper autistic people's performance on ToM tests in general (Tin et al., 2018), and RME performance in particular (Peterson & Miller, 2012).

Study 1 confirmed that the RME can distinguish those with higher levels of autistic traits from those with lower levels. Findings also highlight that those with higher levels of autistic traits underperformed those with lower levels of autistic traits on the RME. Thus, the aim of Study 2 was to determine whether the same pattern of results persisted after replacing photographs of human eyes with matched cartoons. Specifically, the study tested whether those with high levels of autistic traits still underperformed compared to those with low levels of autistic traits when judging the emotions portrayed by cartoon eyes. The design, procedure and materials were identical to Study 1, except the RME was replaced with our adapted cartoon version (CRME).

## Study 2

### Methods

**Figure 1.**
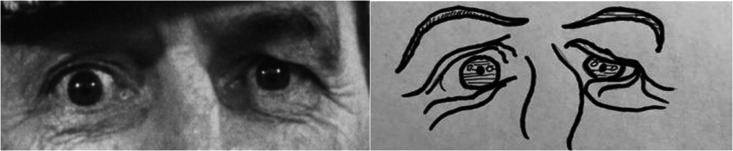
Practices item for the original and adapted RME. The correct emotion is Panicked, presented with the following 3 foils: Jealous, Arrogant and Hatefull.

A professional cartoonist developed cartoon versions of the original 36 RME pictures. The artist was first asked to produce an initial batch of replications in the style of Disney cartoons while keeping the portrayed emotion in line with the original. This full set of 36 drawings were then piloted in line with the initial RME pilot procedure (Baron-Cohen et al., 2001) with the participation of 20 individuals from a different university. This piloting was done online, where each individual saw each of the original RME pictures one by one and had to pick which cartoon version most closely matched the emotion the eyes portrayed. They were explicitly instructed not to weigh spatial and aesthetic features in their decision. The correct drawing was displayed alongside three foils (selected randomly, but with gender kept stable). Pictures which were matched correctly with their cartoon at or above 50% (chance rate would be 25%) were retained (23 items, (mean accuracy rate for these items was .733 SD = .175), while those who had an accuracy rate below 50% (13 items, M = .30 SD = .10) were identified for further development. These 13 items were then redeveloped by the artist and replaced. These 13 new items were repiloted on 20 new participants, in the same way as before. Nine of them were correctly matched with their cartoon drawing above 50% (M = .61 SD = .11) while four still had an accuracy rate below 50% (M = .23 SD = .09). We, therefore, accepted the nine items which were now chosen above chance and retained these for testing. The four which were below chance level were matched less accurately than their original versions from the first pilot. Therefore, we reverted to the original versions, and report analyses for the CRME with all 36 items (Full CRME) and with these four items excluded (Adjusted CRME) in order to have our adapted test include the full range of emotions presented in the original RME. The original photographs in the RME were replaced with this final set of cartoon drawings, while all other aspects of the test were identical to Study 1. The practice item for both the original and our adapted version of the RME can be found in Figure 1. A copy of all original and cartoonized eyes, along with the correct (highlighted) and the three foil emotions they are presented with can be found XX - Authors website where measure is available blinded for review – XX

#### Participants

Students from a university in the Southwest region of the United States as in Study 1 participated in Study 2 (n = 396, 315 females, Mage = 21.91, range 17-67, the ethnicities were: 26.3% White, 33.2% Asian, 21.8 Hispanic, 9.9% Black, 6.1% other, the average level of college attainment was some). Anybody who had previously taken part in Study 1 was excluded from taking part in Study 2. To account for this, and to achieve a similar sample size, the study window was set at two months for this study. This study received approval from the university's Institutional Review Board.

### Results

Data for the AQ (KS(396) = .068, p = < .001), the CRME Full Total (KS(396) = .123, p = < .001), the CRME Adjusted Total (with the 4 items that failed piloting checks removed, KS(396) = .12, p = < .001)), all violated the assumptions of normality. No outliers were excluded, and non-parametric statistics were used. A non-parametric correlation confirmed the expected negative relationship between AQ and RME score for both the Full CRME (rs = −.213, n = 396, p < .001) and the Adjusted CRME (rs = −.216, n = 396, p < .001). This confirms that the cartoon version of the RME was still related to AQ traits in line with Study 1.

The High AQ group was formulated based on individual AQ scores, in line with Study 1. This included 13 participants (3.28% of the total n) scoring within the clinical range. The matched Low AQ group was then formulated by including the 13 participants with the lowest AQ scores. An independent-samples Mann Whitney U test was then performed to explore whether there was a significant difference in CRME scores between the two groups. There was not a significant difference between the High and Low AQ groups on either the Full CRME (U = 110.0, Z = 1.32, p = .19, r = .26). or Adjusted CRME (U = 102.0, Z = .905, p = .39, r = 0.177). See Table 2 for all descriptive statistics. This shows that, as hypothesized, when the RME is presented using cartoon instead of non-human agents, those with high levels of autistic traits no longer underperform in relation to those with low levels of autistic traits

**Table 2.** Descriptive statistics for the AQ total and the full and adjusted CRME proportion correct for Study 2.

		AQ			Full CRME				Adjusted CRME			
Group type	M	SD	Mdn	Range	M	SD	Mdn	Range	M	SD	Mdn	Range
High AQ	33.46	1.71	32.88	32–36	.64	.10	.64	.50–.81	.67	.12	.68	.47–.88
Low AQ	9.69	1.44	10.0	6–11	.70	.10	.69	.58–.92	.71	.10	.71	.56–.91

## General discussion

It was hypothesized that while High AQ individuals would underperform Low AQ individuals on the RME, this would no longer be the case for our anthropomorphic version of the test (CRME). The results confirmed that while those with high levels of autistic traits indeed underperformed on the original RME compared to those with low levels of autistic traits, but they did not significantly differ in their ability to interpret cartoon emotions.

There are several theories as to why this may be. First, it has been suggested that autistic people and those with a high degree of autistic traits may compensate for their difficulties by processing facial emotions through the use of more explicit, rule-based strategies (i.e. a sad mouth turns downwards) (Rutherford & McIntosh, 2007). For instance, they appear to rely more on individual pieces of the face like the mouth in turn rather than focusing on the broad configuration of the face. As the emotions of non-human faces, such as those of the cartoons used in this study, often exhibit 'exaggerated realism' (Thomas et al., 1995), exploiting the clear exaggeration of the cartoon expression (i.e. wide eyes, raised eyebrows, creased brows) to derive rule-based conclusions about the emotional expression may be driving these and others' (Brosnan et al., 2015) findings. Indeed, preliminary research suggested that when the emotional expressions of a social actor were exaggerated, autistic participants were more socially responsiveness (Carter et al., 2016) and they rated exaggerated, cartoon-like expressions as more natural than controls (Rutherford & McIntosh, 2007). As cartoon faces are by their nature 'caricaturized' or exaggerated, it may also increase distinction and thus memorability, an effect shown in response to caricatures in typical development (Kaufmann & Schweinberger, 2012).

Additionally, in line with the social motivation theory (Chevallier et al., 2012), these results may reflect the increased salience of cartoons by autistic people, thus influencing their responsiveness. Indeed, autistic children and adolescents have been shown to spend more time viewing cartoons than any other media (Kuo et al., 2014), and as shown in Silva et al. (2015), they found animated stimuli more engaging than human stimuli, and to a greater extent than controls. Indeed, there may be specific aspects of cartoon stylization that autistic people find particularly rewarding. For instance, cartoon agents are often represented with large eyes (Gould, 2010), which corresponds to the type of cute 'baby face' schema that cues attention (Borgi et al., 2014), increases motivation and neural reward activity (Glocker et al., 2009), and even links with improved performance on tasks like a visual search (Nittono et al., 2012). Thus, face processing differences in autistic populations with regards to human and cartoon representation may reflect differences in motivation to engage with certain types of eyes. The eye-avoidance hypothesis (Tanaka & Sung, 2016) purports that autistic people have an increased sensitivity to eyes which makes direct gaze uncomfortable, and thus decreased attention to eyes is a compensatory mechanism to avoid over-arousal. Researchers Moriuchi et al. (2016) instead suggested that autistic people have an eye-insensitivity, and argued that ASC is an extreme case of diminished social motivation, reflected in a reduced interest in attending to socially-communicative parts of the face.

The present study utilized a between rather than within-subjects design, in that separate samples were tested on either the original or cartoon RME. This was done as retesting individuals on the RME would introduce practice effects. Equally, presenting stimuli in both cartoon and human form may confound results as some emotion recognition questions may be more challenging than others, and motivation stemming from one presentation form may carry-over to another. That is, increased engagement from a given cartoon item may impact a subsequent non-cartoon item. However, a within-groups design would have allowed a more direct comparison of differences between cartoon and human versions of the stimulus. Indeed, work by Cross et al. (2019) showed that young autistic children were significantly better at recognizing emotions from full-face pictures when they were presented in anthropomorphic filters. Finally, this study utilized a heavily female sample. While previous work has shown the RME to be resilient to sex differences, in order to check this additional unplanned analysis was undertaken in order to rule out that RME performance differed significantly across males/females. This further analyses, which can be found in Supplemental Material, showed there was no significant difference between males/females on either RME or CRME performance.

In conclusion, we showed that while the RME reliably finds that people with high degrees of autistic traits perform less well at emotion recognition than those with lower levels of autistic traits, High and Low AQ groups did not differ on a cartoonized version of the RME. While several studies (see Atherton & Cross, 2018 for a review) have found that presenting autistic samples with non-human rather than human faces can improve FER, this study shows this effect in adults from the general population with higher levels of autistic traits using a well-established test that explicitly tests eye FER.

Future research should explore these underlying mechanisms affecting the cartoon RME by measuring eye-gaze patterns with original and cartoon versions of the task. It will also be of interest to conduct brain imaging assessments on autistic samples using cartoon versions of tasks such as the RME, other well-established FER tests, as well as dynamic cartoon FER assessments that could be a proxy for real-life social engagement. For instance, Chaminade et al. (2015) measured brain activation in autistic and non-autistic people during a game of ‘paper, rock, scissors’ they played against human, robot and computer opponents. They found a lack of social motivation signalling in all conditions for autistic participants, while non-autistic participants experienced signalling only when playing against a human opponent. However, in a preliminary study by Carter et al. (2016) autistic children were shown to be more socially responsive to social actors when they were cartoon rather than human-like. Assessing whether there are differences in neural activation when autistic people interact with a cartoonized agent, particularly in areas of the brain related to social motivation, would be of interest.

There are some clinical implications of this research that enhance the relevance of this study. First, with relation to the CRME, this study suggests that just like child samples studied previously, adults with high autistic traits are also compensating for FER deficits when engaging with stimuli that is cartoon. While historically cartoons have been viewed as children’s media, there is now a abundance of cartoon entertainment marketed explicitly for adults. Thus, it may be that engagement with cartoon media is something that adults with autism and high autistic traits may not only particularly enjoy, but they may find such media more engaging and easier to understand. Rozema (2015), for instance, wrote about his autistic son’s experiences with Japanese manga and how anime may be particularly salient and digestible for those on the spectrum.

Parents, educators and mental health professionals may want to explore ways they can connect with the autistic people in their lives through cartoons, and encourage this type of medium, understanding that it may enhance emotion recognition in those on the spectrum. Indeed, autistic people and those with high autistic traits should also be encouraged to use the medium to express themselves. For instance, Martin et al. (2019) detailed the co-creation of a comic intervention for autistic children with autistic children to teach social constructs, and Birge (2009) discussed several comics made by parents of autistic children that teach about the condition in a way that is humorous and accessible to all. This underscores the importance of engaging with and teaching through mediums such as cartoons that may particularly resonate with people on the spectrum.
